# Current trends in tendinopathy: consensus of the ESSKA basic science committee. Part I: biology, biomechanics, anatomy and an exercise-based approach

**DOI:** 10.1186/s40634-017-0092-6

**Published:** 2017-05-30

**Authors:** F. Abat, H. Alfredson, M. Cucchiarini, H. Madry, A. Marmotti, C. Mouton, J.M. Oliveira, H. Pereira, G. M. Peretti, D. Romero-Rodriguez, C. Spang, J. Stephen, C. J. A. van Bergen, L. de Girolamo

**Affiliations:** 1 Department of Orthopaedic Sports Medicine, ReSport Clinic, Passeig Fabra i Puig 47, 08030 Barcelona, Spain; 2 Sports Medicine Unit, University of Umeå, Umeå, Sweden; 3 Alfredson Tendon Clinic Inc, Umeå, Sweden; 4 Pure Sports Medicine Clinic, ISEH, UCLH, London, UK; 5 Molecular Biology, Center of Experimental Orthopaedics, Saarland University Medical Center, Kirrbergerstr Bldg 37, 66421 Homburg/Saar, Germany; 6 Lehrstuhl für Experimentelle Orthopädie und Arthroseforschung, Universität des Saarlandes, Gebäude 37, Kirrbergerstr 1, 66421 Homburg, Germany; 7 Department of Orthopaedics and Traumatology, San Luigi Gonzaga Hospital, Orbassano, University of Turin, Turin, Italy; 8 Department of Orthopedic Surgery, Clinique d’Eich-Centre Hospitalier de Luxembourg, 76, rue d’Eich, L-1460 Luxembourg, Luxembourg; 9 3B’s Research Group – Biomaterials, Biodegradables and Biomimetics, University of Minho, Headquarters of the European Institute of Excellence on Tissue Engineering and Regenerative Medicine, AvePark, Zona Industrial da Gandra, 4805-017 Barco, GMR Portugal; 10 ICVS/3B’s - PT Government Associate Laboratory, Braga Guimarães, Portugal; 11 3B’s Research Group University of Minho, ICVS/3B’s–PT Government Associate Laboratory, Braga Guimarães, Portugal; 12 Orthopedic Department Centro Hospitalar Póvoa de Varzim, Vila do Conde, Portugal; 13 Ripoll y De Prado Sports Clinic – FIFA Medical Centre of Excellence, Murcia, Madrid Spain; 14 IRCCS Istituto Ortopedico Galeazzi, Department of Biomedical Sciences for Health, University of Milan, Milan, Italy; 15 Department of Physical Therapy and Sports Rehabilitation, ReSport Clinic Barcelona, Barcelona, Spain; 16 EUSES Sports Science, University of Girona, Girona, Spain; 17 Department of Integrative Medical Biology, Anatomy Section, Umeå University, Umeå, Sweden; 18 Fortius Clinic, 17 Fitzhardinge St, London, W1H 6EQ UK; 19 The Biomechanics Group, Department of Mechanical Engineering, Imperial College, London, UK; 20 Department of Orthopedic Surgery, Amphia Hospital Breda, Breda, The Netherlands; 21 Orthopaedic Biotechnology Laboratory, Galeazzi Orthopaedic Institute, Milan, Italy

**Keywords:** Tendon, Tendinopathy, Consensus, ESSKA, Treatment, Eccentric

## Abstract

Chronic tendinopathies represent a major problem in the clinical practice of sports orthopaedic surgeons, sports doctors and other health professionals involved in the treatment of athletes and patients that perform repetitive actions. The lack of consensus relative to the diagnostic tools and treatment modalities represents a management dilemma for these professionals. With this review, the purpose of the ESSKA Basic Science Committee is to establish guidelines for understanding, diagnosing and treating this complex pathology.

## Review

Despite the physiological adaptation of the tendons to different loads (Langberg et al. 2007), tendinopathy is a clinical problem of great magnitude and is growing in terms of prevalence (Maffulli et al. 2003). Tendon injuries represent approximately 50% of all sports injuries (Maffulli et al. 2003; Andarawis et al. 2015). Several publications (Alfredson and Lorentzon 2002; Cook et al. 2000) have suggested that most pathological disorders involving tendons are mainly degenerative (tendinosis). This is reflected in the presence of non-acute inflammatory cells, the presence of areas of collagen degeneration, myxoid degeneration and an increase in ground substance (Alfredson and Lorentzon 2002; Cook et al. 2000). All these reflect a failure of the native tendon repair process. For these reasons, the enhancement of basic science research and knowledge of the developments in tissue engineering and regenerative medicine (TERM) represent promising fields aiming to find new answers to the treatment of these lesions (Atesok et al. 2012; Pereira et al. 2016).

Tendinopathy is characterized by prolonged pain and is often activity related. Different findings such as tendinosis, paratendonitis, calcifications or partial ruptures are often found in the same tendon (Cook and Purdam 2009). This suggests that, most probably, there is not a single aetiology or a single pathogenesis that is able to explain tendon pain.

Most tendinopathies in the lower limbs involve the Achilles or patellar tendons (Scott and Ashe 2006; Sobhani et al. 2013). The most frequent tendinopathy in the upper limbs is lateral epicondylitis or enthesopathy of the extensor carpi radialis brevis (Scott and Ashe 2006).

In general, the term “tendinopathy” refers to a pathological condition of a tendon with a complaint of pain and swelling (van Dijk et al. 2011). Tendinitis reflects acute tendon injuries that are characteristically combined with inflammation and the presence of acute inflammatory cells and proteins. On the other hand, tendinosis, which is much more commonly found in the Achilles, suggests a chronic tendon lesion with damage to the tendon on a cellular level. This damage is characterized by collagen bundle disorganization, an increase in ground substance, a high number of tenocyte nuclei, hypoxia and the presence of lipid vacuoles. The injured tendon often has vascular infiltrations of small blood vessels and small nerve ingrowth (Xu and Murrell 2008).

In those tendons covered by a synovial sheath, the pathology at this level is called tenosynovitis. However, the Achilles tendon or Patellar tendon does not have a synovial envelope but is instead covered by paratenon. Conversely, pathological changes at this level are referred to as paratendinopathy, acute or chronic (van Dijk et al. 2011).

Diagnostic ultrasound (US) in tendinopathies is the most appropriate and advantageous imaging modality for routine clinical evaluation (Warden et al. 2007; Roy et al. 2015). A US-guided technique has demonstrated more accuracy rate than the landmark-guided technique in the greater part of the joints and tendons (Kane and Koski 2016). Therefore, the use of US imaging in the diagnosis and treatment of tendinopathies should be mandatory.

This article presents the consensus of the ESSKA Basic Science Committee for a better understanding, diagnosis and treatment of tendinopathies. Part I is focused on the conceptual knowledge of tendinopathies, anatomy, biomechanics and an exercise based approach while part II is focused on treatment options.

## Biomechanics

The aetiology of tendinopathy is multifactorial, with an unclear pathogenesis. However, mechanical loading and, more specifically, alterations to regular loading are widely recognised to play a critical role in its onset and perpetuation (Roberts and Konow 2013). As the outlined tendons are composed of collagen fibrils, allowing energy storage, they have evolved primarily to transmit tensile load and reduce the rate of force transmission to the attached muscle (Roberts and Konow 2013). Fatigue in response to mechanical loading has been seen. These adaptations are thought to make tendons susceptible to pathological changes (Andarawis-Puri and Flatow 2011).

### Mechanical behaviour

**Fig. 1:**
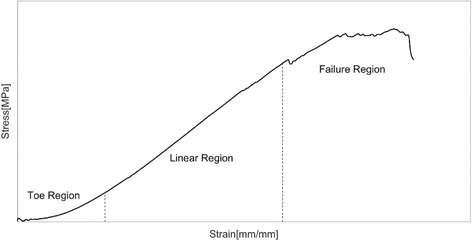
An example of an Achilles tendon loaded to failure in isolation, displaying three distinct regions in response to the tensile loading

Traditionally, the mechanical properties of tendons have been evaluated and reported through the use of tensile testing methodologies in vitro (Benedict et al. 1968). These methods can be used to characterise the mechanical response of the tendon and are typically presented as stress-strain curves (Fig. 1). These curves display three typical regions. Namely, they are 1.) the toe region where the sample is tensed typically to approximately 2% of strain (sufficient to remove any resting fibre crimp, but not to cause any structural damage), 2.) the linear region where loading causes elongation of fibers typically at approximately 4–8% of strain, where some fibre damage will begin (microscopic failure) and 3.) the failure region where complete breakage of all fibres occurs (macroscopic failure) at approximately >12%.

A normal tendon shows regular banding related to its waveform configuration (crimps). When the tendon is loaded, the force-displacement curve shows a distinct toe region with a decrease in crimping and an increase in collagen alignment (Franchi et al. 2007).

In vivo strains remain difficult to accurately quantify despite the use of various implantable devices. However, there is thought to be a large gap between the tendon strains reported in vivo and those experienced during typical daily activities, usually less than 4% (Maganaris and Paul 1999). Therefore, rather than maximal loading, it is thought that repetitive loading at lower levels causes damage to the collagen fibrils and/or fibres, resulting in a reduction of the tendon cross sectional area over which muscular forces are transmitted, making them more susceptible to failure.

Tendons are recognised as transmitting longitudinal tensile loads from muscle to bone. However, since tendons commonly wrap and twist, they can also be subjected to compressive, shear and transverse forces (Vogel 2003). Interestingly, tendon pathology is predominately found on the joint side of the enthesis, e.g. the calcaneal side with the Achilles tendon (Rufai et al. 1995) and on the humeral side of the supraspinatus (Andrews et al. 1985). Researchers have found that strains near the insertion site within tendons are not uniform, with lower strains commonly identified on the joint side (Almekinders et al. 2002). That is the opposite of what might be expected. This has led to the proposal that the area of tendon afflicted by tendinopathy may be stress shielded, undergoing degenerative changes as opposed to the common theory of overload. Alternatively, it has been found that there is a significant compressive force acting on the patellar tendon as the inferior pole of the patella impinges the tendon (Basso et al. 2002), suggesting compressive force may play a more significant role in tendinopathy. Investigations into the tibialis posterior tendon report that cartilaginous metaplasia can occur as an adaptive response to mechanical compression on the tendon against the medial malleolus (Vogel et al. 1993). Clinically, the compression model provides a rationale for why patients with insertional tendinopathies may dislike compressive joint patterns such as loading in ankle dorsi flexion that is often observed in insertional Achilles tendinopathy populations (Cook and Purdam 2012).

Numerous factors are reported to alter the mechanical response of tendons and thus play a role in tendinopathy. Overuse injuries, including tendinopathy, are found to be more common in elderly athletic populations compared with younger ones (Fahlström et al. 2002). From the third decade onwards, cellular function in tissue declines and tendons are subjected to degenerative changes, making the aged tendon weaker and thus more susceptible to overuse (Kannus et al. 2005). Thus, tendinopathy has been strongly correlated to aging. Disuse is also identified as altering tendon properties. Denervation and immobilisation have both been found to decrease tendon stiffness and reduce final tissue strength (Savolainen et al.^1988^; Loitz et al.^1989^). Conversely, an increase in physical activity is found to improve tendon material properties such as Young’s modulus as a result of alterations to the collagen make-up (Butler et al. 1978). The Young’s modulus of a tendon (slope of the stress-strain curve) is an important contributor to whole muscle short-range stiffness, playing an important role in body stability.

There is substantial evidence to support the relationship of mechanical loading to the onset and perpetuation of tendinopathy as a result of alterations to typical loading patterns, inappropriate loading volume or frequency resulting in tissue overloading or underloading. A full understanding of the biomechanical basis for tendinopathy is still lacking and continues to evolve.

## Anatomy and classifications

### Patellar tendinopathy

Patellar tendinopathy is a frequent degenerative load-induced injury (Hägglund et al. 2011; Cook and Purdam 2009). It is characterized by progressive patellar tendon pain, tenderness to palpation in combination with anterior knee pain that leads to recurrent or long-standing impairment of athletic performance (Lian et al. 2005).

Different prevalence rates for this injury in the athletic population have been reported. Lian et al. (2005) reported a prevalence of 45% in volleyball players, 32% in basketball players. Durcan et al. (2014) showed that 9% of the rugby players developed patellar tendinopathy during a season. Zwerver et al. (2011) and Hägglund et al. (2011) reported a higher prevalence rate (12–27%) in top athletes.

Different factors influence the timing of patellar tendinopathy. Among the intrinsic, are factors such as an imbalance of force, postural misalignment, foot shape and type of footfall, the mobility of the ankle and a deficit of strength or flexibility in the lower extremities (Witvrouw et al. 2001). The extrinsic factors include improper training surfaces, inappropriate training equipment, excessive loading, high intensity training or repetitive loading (Hägglund et al. 2011; Durcan et al. 2014).

**Fig. 2:**
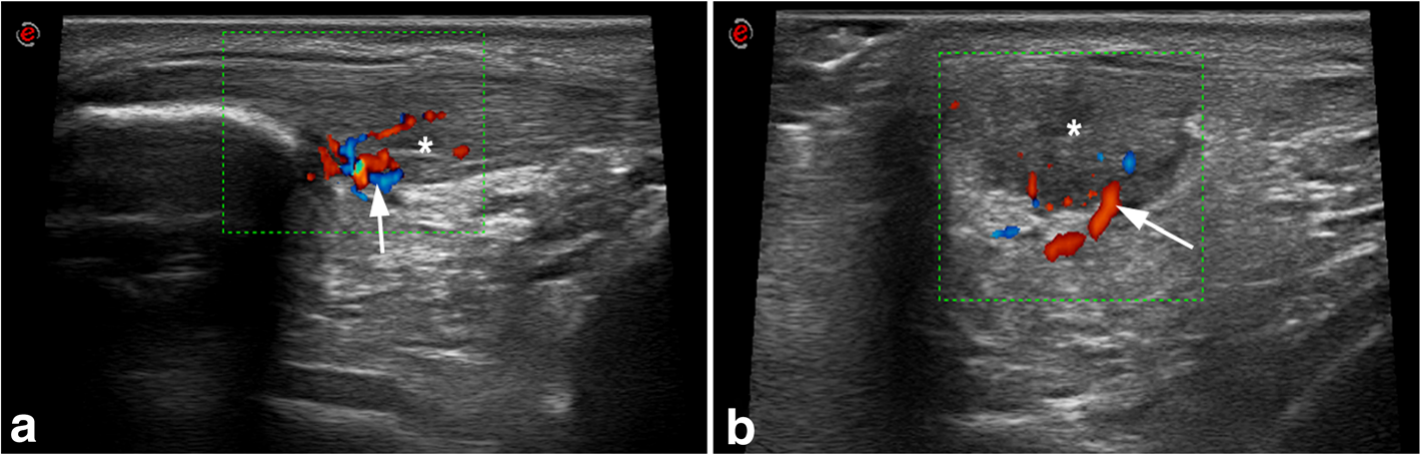
Insertional patellar tendinopathy confirmed with high definition gray-scale US. Longitudinal (**a**) and transversal view (**b**) of an injured patellar tendon. The proximal patellar tendon has hypoechoic zones (*) and the color-doppler analysis shows intensive hypervascularization (*arrow*)

An US examination (Fig. 2) clearly shows intra and peritendinous changes with collagen disorganization. The clinician can find thickness of the tendon and different degrees of hypoechogenicity (Comin et al. 2013). In some cases, collagen lesions can be found in combination with neovascularization and degenerative changes, more frequently at the bone insertion and in the mid-substance of the tendon. Some authors have reported some hypoechoic regions inside the tendon and spindle-shaped thickening in elite asymptomatic soccer players (Fredberg et al. 2008). Studies on other types of athletes noted the same findings (Comin et al. 2013).

Patellar tendinopathy can be classified in accordance with the localization of the injury. The most frequently affected zone is the inferior pole of the patella, in the deeper part of the tendon (Malliaras et al. 2015). This zone represents the area of maximum tension during exercise. Equally, patellar tendinopathy can affect the midportion zone or insertion at the tibial tuberosity (Sarimo et al.^2007^). Irritation of the infrapatellar bursa at the distal part of the patellar tendon, in his insertion in the tibial tuberosity, often coexists with distal patellar tendinopathy (Benjamin et al. 2004).

There is a relationship between the alteration of the Hoffa fat pad (oedema and inflammation) and patellar tendinopathy (Dragoo et al. 2012). An alteration of the fat pad must be correctly diagnosed to apply the appropriate therapeutic protocol (Ward et al. 2016).

### Achilles tendinopathy

Since the 17th century, the “tendo magnus of Hippocrates” has been referred as the Achilles tendon. Throughout the years, many classifications and eponyms have been used to describe its disorders, diagnostic tests or anatomic structures in the hindfoot related to its pathological conditions (van Dijk et al. 2011). Such pathologies have relevant and frequent implications in both high-level athletes and the general population.

Generally patients complain of pain and swelling which limit activity. Chronic Achilles tendon pathologies are among the most common injuries in sports comprising running and repetitive jumping. In elite long-distance runners, there is a 52% risk of having an Achilles tendon injury at least once (Kujala et al. 2005). An incidence of 3% with a recurrence rate as high as 27% has been reported in soccer players (Gajhede-Knudsen et al. 2013). However, 30% of the patients suffering from Achilles related conditions have a sedentary lifestyle (Ames et al. 2008).

The origin of Achilles tendinopathy is multifactorial with intrinsic factors like dysmetria or misalignment of the lower extremities, ankle mobility deficits or forefoot deformities playing a significant role. Extrinsic factors also influence mechanical overload or overuse of the tendon. Other factors such as advanced age, diabetes or rheumatic diseases also seem to have some relationship to it.

**Fig. 3:**
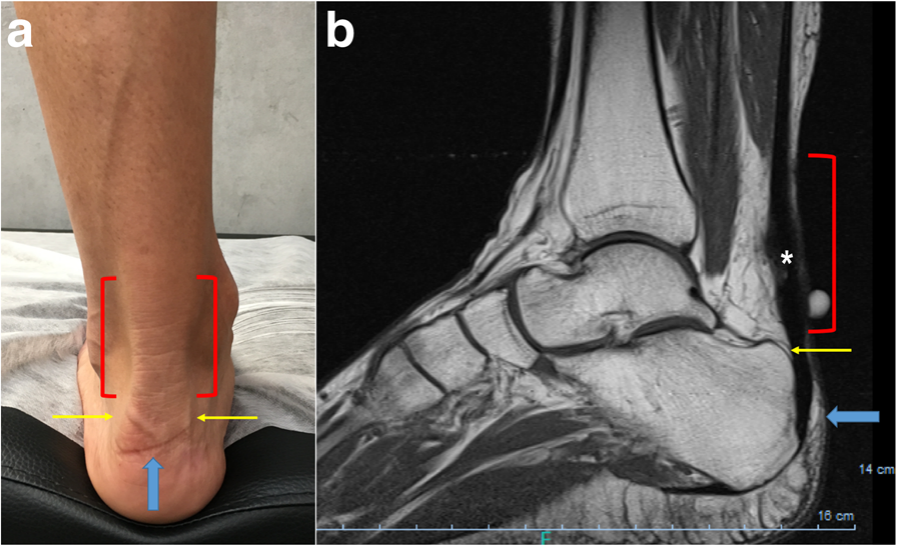
Site of clinical complaints (**a**) and MRI (**b**) correlation of most relevant Achilles pathology sites. Mid-portion Achilles tendinopathy (*red brackets*); retrocalcaneal bursitis (*yellow arrow*); insertional achilles tendinopathy and superficial bursitis (*blue arrow*). MRI sagittal view (**b**) demonstrating Achilles tendon enlargement and signal changings (*)

Recently, an effort has been made to organize and clarify the terminology for Achilles tendon (AT) pathologies in consideration of the anatomic location (Fig. 3), symptoms, clinical findings and histopathology (van Dijk et al. 2011). This classification also considers characteristic findings in different imaging modalities (radiography, US, CT and MRI).

Mid-portion Achilles tendinopathy typically involves an area at 2 to 7 cm from the distal insertion. Patients complain of pain and swelling which limit activity. Degenerative changes of the tendon can be asymptomatic. A deviation of the soft tissue contour can be noticed on plain x-rays. Ultra-sound (US) might show an enlarged tendon with or without fibrillation or hypoechoic areas. Sometimes calcifications are present and can also be seen in CT. An MRI will show enlargement and signal changes in the affected area. It has been hypothesized that the plantaris tendon might play a role in some cases of mid-portion tendinopathy or chronic paratendinopahy in which changes are mainly observed on the medial and ventral area of the Achilles. This might be due to adhesions of the plantaris to the paratenon or tendon as a possible cause of symptoms (van Sterkenburg et al. 2011; Alfredson 2011; Spang 2015).

Achilles paratendinopathy (acute or chronic) usually affects the midportion of the AT. In acute cases of paratendinopathy, a normal AT can be observed in combination with peripheral paratendon enlargement observed in US or MRI. Edema and hyperemia are present and usually aggravated by increased activity. In chronic cases, some changes in the AT might also be noticed as well as peripheral adhesions between AT and paratenon. Patients complain of pain after activity with less swelling after efforts than in the acute setting.

Insertional Achilles tendinopathy at the calcaneus site is usually connected to spurs and calcifications within the tendon. Patients refer to pain, some stiffness and very often to a solid permanent swelling. That pathology is denominated enthesopathy. Small degenerative ruptures of AT might be present. Bone spurs can be identified in x-rays, CT scans, US or an MRI (which also might show some tendon signal changes). CT is very important to planning once it provides the exact locations of bony spurs or calcifications.

Changes located in the bursa in the retrocalcaneal recess between the anterior inferior side of the AT and the posterosuperior aspect of the calcaneus are known as *retrocalcaneal bursitis*. There is painful soft tissue swelling at this level, both medially and laterally to the AT. Hypertrophy of the bursa with synovial infoldings and accumulation of fluid are observed either as primary arthropathy or secondary to local recurrent conflict. An x-ray might show radio-opacity of the retrocalcaneal recess and sometimes deviation of soft tissue contours (van Dijk et al. 2011). US might identify fluid in the retrocalcaneal bursa while an MRI is expected to demonstrate intense signal on T2 images.

Superficial calcaneal bursitis involves the bursa located between the skin and AT presenting a painful, solid swelling on the calcaneus (more often postero-lateral) and is connected to shoe wear. An x-ray might show changes in soft tissue, while US confirms fluid in that location. An MRI confirms signal changes but is rarely required.

Finally, a rupture of the AT should be considered as a different topic to the formerly described AT pathology. Most often, such ruptures occur in a non-contact mechanism and is associated with progressive degenerative changes of the AT (Carmont et al. 2013). This possibly devastating condition, particularly in high-level athletes, is also linked to the impairment of native repair mechanisms to defend the tendon from degeneration and ultimately rupture. Several attempts have been made to develop new biological options to diminish the risk of AT failure and enhance the repair strategies to arrive at faster healing and strong tissue (Carmont et al. 2013).

### Rotator cuff tendinopathy

Rotator cuff (RC) tendinopathies are present in around 30% of the general population, being the most common form of shoulder pain. (Alqunaee et al. 2012). Quality of life (QoL) in patients with RC tendinopathy is significantly affected with functional limitation, decreased range of motion (especially external rotation and elevation) and strength, pain and the inability to perform overhead actions (Bodin et al. 2012).

Inside the general term of RC tendinopathy, we can include tendinitis, tendinosis, paratendinosis or partial tears of the RC tendons. We shall exclude subacromial impingement syndrome, subdeltoid bursitis, adhesive capsulitis and full thickness tears (Hanratty et al. 2012).

The affected RC muscles are the supraspinatus, infraspinatus and teres minor in the posterior part of the shoulder and the subscapularis in the anterior part. They provide internal and external rotation torque (Boettcher et al. 2009). These muscles also provide shoulder joint stability during different movements.

Different mechanisms have been proposed as causal agents of RC tendinopathy. Extrinsic mechanisms should include attrition of the tendons with bony structures like the humeral head or acromion, probably due to fatigue, weakness, pain or muscular incompetence (Neer 1983). Approximately half of the patients suffer subacromial space reduction (Chopp et al. 2010) that can be normalized with a detailed rehabilitation and exercise protocol (Savoie et al. 2015). Intrinsic factors can include age, genetics, altered biomechanics and vascular or inflammatory changes (Lewis 2010). Despite all of this, the most important factor is the excessive tissue load because RC tendinopathy generally occurs in the dominant limb in sports or work occupations (Yamamoto et al. 2010; Sein et al. 2010; Bodin et al. 2012).

The best diagnostic tool for RC tendinopathies is a US image followed by an MRI. A correct and precise clinical history and examination will determine the need of further examinations. For example, to evaluate bony lesions an MRI is more accurate. However, MRI arthrography is mandatory in cases of labral or capsular lesions (Pavic et al. 2013; Rutten et al. 2010). The differential diagnose of all the shoulder structures will determine the best treatment option.

### Lateral epicondylitis

Lateral epicondylitis is also generally known as tennis elbow. The term epicondylitis is actually confusing. It implies that inflammation is present at the lateral humeral epicondyle. The fact is that inflammation is only present in the very early stages of the disease and the exact anatomic localization of the pathology is not the lateral epicondyle (Brummel et al. 2014). Over the years, it has become clearer that the source of the condition is situated at the origin of the extensor carpi radialis brevis (ECRB) (Boyd and McLeod 1973; Kraushaar and Nirschl 1999). Thus, “ECRB tendinosis/enthesopathy” or “lateral elbow tendinopathy” might be a more accurate term to describe the disease (Ali and Lehman 2009; Gregory et al. 2016).

Numerous investigations have aimed to identify the exact underlying pathological mechanism that causes the disease. Microscopic and macroscopic tears of the common extensor tendon have been proposed as the underlying cause. Furthermore, various studies have found a degenerative tendinopathy as well as granulation tissue at the ECRB origin as a result of repetitive microtrauma (Brummel et al. 2014).

The histological findings related to lateral epicondylitis have been described as angiofibroblastic hyperplasia and angiofibroblastic tendinosis (Nirschl 1992). These findings are characterized by disorganized and immature collagen and fibroblastic response. Furthermore, increased apoptosis and cellular autophagy of the tenocytes that resulted in a disrupted collagen matrix and weakening of the tendon have been observed (Chen et al. 2010).

Lateral epicondylitis is a very common disorder. It occurs not only in tennis players but also in the general population. The annual incidence in general practice is 4–7/1000 people (Buchbinder et al. 2010). Its prevalence stands at 1 to 3% in the general population and 7% in manual labourers (Gregory et al. 2016). The most common age group is 42 to 54 years (Buchbinder et al. 2010, Shiri et al. 2006). In one large study, men and women were equally affected (Shiri et al. 2006) while females were affected more than males in another study (Buchbinder et al. 2008)

The patient typically presents with pain at the lateral aspect of the elbow. Grasping objects often exacerbates the pain. On physical examination, there is tenderness at the origin of the ECRB and lateral epicondyle of the humerus. The pain is provoked by extension of the wrist against resistance. However, the value of physical examination is limited in diagnosing lateral epicondylitis (Kryger et al. 2007). The differential diagnosis includes osteochondritis dissecans of the capitellum, radiocapitellar osteo-arthritis, varus instability or posterolateral rotatory instability and radial tunnel syndrome.

Classification systems for lateral epicondylitis are scarce in the literature. Some authors differentiate between the extra-articular type, intra-articular type and the mixed type (Zhu et al. 2013). Others have made a classification model to assist physiotherapists in identifying appropriate treatment options (Wixom and Lastayo 2012). This classification model stratifies patients as “severe”, “moderate”, or “mild” based on signs and symptoms, pain, range of motion, resistance tests, grip strength and tenderness. However, none of the classification systems has been shown to predict treatment outcomes.

## Exercise based approach

For more than 30 years, eccentric exercise has been presented as one of the best options to treat chronic tendinopathy. Komi discussed about the need to introduce this kind of exercise when treating these injuries (Komi 1979). Then, Stanish suggested a protocol to treat chronic patellar tendinopathy centred on eccentric training (Stanish et al. 1986). Cook et al. (1997), in a retrospective study with athletes suffering from jumper’s knee, referenced the work by Stanish (1986) that indicated eccentric work after doing concentric exercises. Some years later, Alfredson (in the first published study on a patient material) created a successful protocol of eccentric training of calf muscles to treat chronic Achilles tendinopathy (Alfredson et al. 1998). Coming after all those works and other original published research in this field, several reviews have since described good results from eccentric training in chronic tendinopathy (Kingma et al. 2001, Wasielewski and Kotsko 2007; Visnes and Bahr 2007; Meyer et al. 2009).

Per these publications, eccentric work has become the go-to conservative treatment option for chronic tendinopathies (Duthon et al. 2012; Rodríguez-Merchan 2013; Murtaugh and Ihm 2013). Relative to specific locations, eccentric training shows exceptionally good results when applied to both chronic Achilles and patellar tendinopathies (Visnes and Bahr 2007; Woodley et al. 2007; Meyer et al. 2009; Maffulli et al. 2010; Gual et al. 2016). Other reviews in the literature also support eccentric training for lateral epicondylar tendinopathy and the tendons of the shoulder rotator cuff (Murtaugh and Ihm 2013; Camargo et al. 2014). However, there is a significant lack of evidence that confirms eccentric training having clear positive results in elbow and shoulder tendon injuries.

Although eccentric training is confirmed to be beneficial in the treatment of chronic tendinopathies, several issues relative to training programs are yet to be clarified.

In terms of the possibility of isolated eccentric training (decreasing or eliminating concentric actions from the stretch-shortening cycle, SSC), there is no strong evidence to consider this possibility as a good option when treating chronic tendinopathies (Couppé et al. 2015; Malliaras et. 2013).

When comparing isolated actions, eccentric training shows better results than the application of concentric work. These benefits have been shown in Achilles (Mafi et al. 2001), patellar (Jonsson and Alfredson 2005) and tennis elbow tendinopathies (Peterson et al. 2014).

More recently, the Van Ark and Rio (2016 and Rio et al. 2017) investigated the introduction of isometric actions to jumping athletes suffering from patellar tendinopathy. Those studies show good results in pain reduction but more investigation is needed to understand whether and when to apply isometric work to chronic tendinopathy.

The most extensive eccentric protocol to treat chronic tendinopathy was published by Alfredson et al. (1998). It combined high frequency-low intensity eccentric training on a decline board that showed great positive results. Since publication, the protocol has been replicated in several studies. Purdam et al. (2004) compared the same exercise performed on the decline board and directly on the ground in subjects with patellar tendinopathy. That comparison demonstrated pain reduction only when performing on the decline board and no effects when working without the board. The study was replicated by Young et al. (2005) in volleyball players and it found that both groups saw decreased pain intensity (VAS) and increased functionality (Victorian Institute of Sport Assessment for Patella: VISA-P) with little difference in favour of the decline board. Based on these studies, we can state that the most important variable to bring about positive effects was eccentric training even though Visnes et al. (2005) did not find positive effects by applying Alfredson’s protocol (the differing methodological aspects could explain this result) in volleyball players with patellar tendinopathy. Years after Biernat et al. (2014) applied the same protocol, also with volleyball players, with the addition of an unstable surface on the decline board. The result was a decrease in pain in cases of patellar tendinopathy. Alfredson’s protocol has also shown positive effects in the circulatory aspects of chronic Achilles tendinopathy (Knobloch et al. 2007), not only in mid-portion injuries but also at the insertional level of the tendon with little variation in the eccentric execution (Jonsson et al. 2008). All these positive conclusions have also been described by the reviews of Gaida & Cook (2011) as well as Habets & van Cingel (2015). Nevertheless, there is a weak point in these studies. It is the poor follow-up developed with positive results in some cases (Gärdin et al. 2010) and relatively negative ones in others (van der Plas et al. 2012). It is important to specify that this protocol is not sport specific in any case, which represents a limitation relative to the right return-to-play process sport players need to follow.

Continuing with different methodological options to treat chronic tendinopathy, Frohm et al. (2007) compared Heavy Slow Resistance Training (HSRT) with the Bronsmans device, which consists of high intensity work, to the Alfredson’s protocol in chronic patellar tendinopathy. Both groups showed improvements in VAS and VISA scores without significant differences between them. Kongsgaard et al. (2009) compared the effects of corticosteroid injections, eccentric decline squat training and heavy slow resistance training in subjects with chronic patellar tendinopathy. Only the two training groups registered good improvement in the long term, there being better results in the HSRT group. In spite of the aforementioned published works, the review by Larsson et al. (2012) advocated for Alfredson’s protocol as the first option to treat chronic patellar tendinopathy, only recognizing moderate evidence for the use of HSRT. More recent reviews (Kjaer and Heinemeier 2014; Pearson and Hussain 2014) have highlighted the good results of eccentric work with both Alfredson’s protocol and the HSR training. Beyer et al. (2015) registered good results with both kinds of training in mid-portion Achilles tendinopathy but patient satisfaction was greater after working with HSRT.

**Fig. 4:**
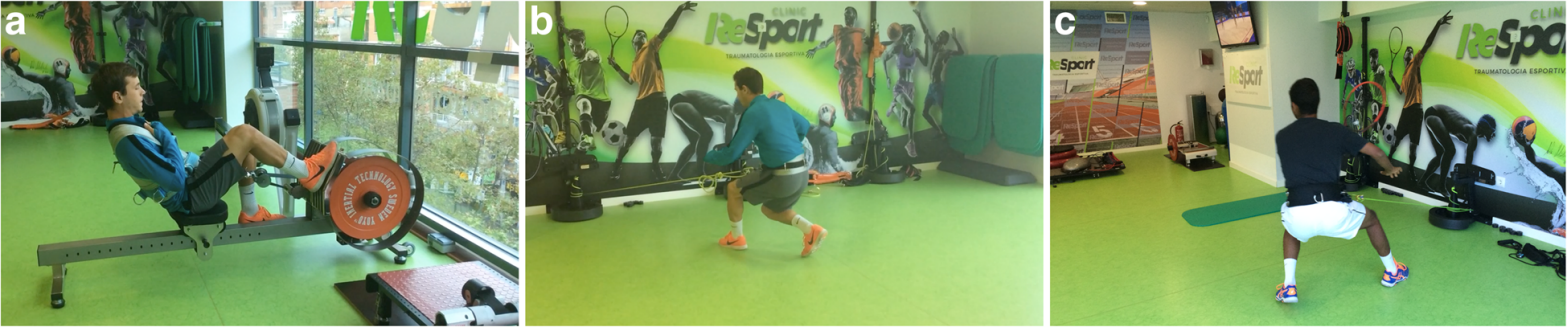
Exercises for a sports player suffering from tendinopathy must introduce actions from general orientation (**a**) to more functional tasks (**b** and **c**). In all these exercises, the load is more concentrated on the patellar tendon, working unilaterally with inertial resistance to emphasize in the eccentric work and the eccentric – concentric transition as well

Another high intensity training modality (Fig. 4) has been applied with isoinertial resistance devices that emphasize eccentric actions to treat chronic patellar tendinopathy in athletes (Romero-Rodriguez et al. 2011). This study registered improvement in VAS and VISA scores and an increase in quadriceps strength. Inertial resistance has also been studied in combination with medical techniques like USGET (Abat et al. 2015). It offered excellent results (VISA and Tegner scales) in the treatment of insertional chronic patellar tendinopathy in athletes.

Although the analysed works with different methodologies highlight the effectiveness of eccentric work for chronic tendinopathy, a problem arises from the first publications. It is the lack of consensus relative to the optimal load, frequency and recovery periods between training sessions (Larsson et al. 2012; Pearson and Hussain 2014; Couppé et al. 2015; Habets and van Cingel 2015). Some possible reasons for the lack of consensus are the different anatomical locations studied, the quality of the studies and the lack of follow-up information.

## Conclusions and challenges

The question put forth by Purdam et al. (2004) is of significant relevance when treating chronic tendinopathy. We are slightly modifying this question to ask whether most of the published works are offering strength training or not. Might these interventions be more identified by an active stretching program with relative low-intensity eccentric actions? It looks like the published works do not offer strength interventions and it might be that only inertial resistance and heavy load resistance training develop the minimum level of tension to identify them as strength training, at least in sport players.

Related to the previous point and in accordance with the articles cited, isolating the eccentric actions to a greater or lesser extent is useful when facing the first stage of an injury. However, the complete execution of the stretch-shortening cycle will be necessary to adapt the tendon to a higher intensity in athletes and in the daily activities of subjects who are not athletes.

In consideration of the first two points of these conclusions, specificity of interventions will be needed when speaking about long-term effects. In sport, it is known how the behaviour of the tendon varies with the speed of the load applied to it. With more elastic actions, the tendons will be stretched for a longer period and will present more deformation but faster actions will have a more reactive profile and the tendon will face greater loads over very short periods. These situations are especially present when developing sports skills. The question arises when the studies presenting a follow-up have not developed a sport-specific return-to-play process to complete the needs of the tendon in competition. If the published studies stop their interventions with general exercises, the follow-ups will have a limited interpretation.

If we consider specific interventions, specific assessment will also be needed. To assess the evolution of a subject with tendinopathy with different subjective scales is a good tool but an assessment of muscle power and strength, dynamic balance, speed and other more representative parameters of sports skills will be needed.

**Fig. 5:**
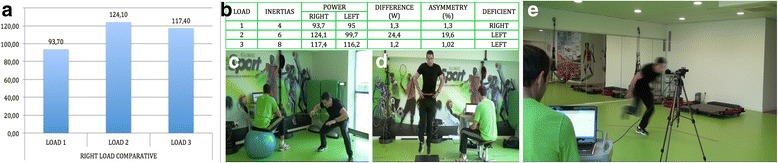
The assessment of the therapeutic and return to play processes must be as specific as possible. Figures (**a**) and (**b**) shows the study of leg asymmetries in a change of direction task (**c**). Jumping action with a contact platform (**d**) and assessment of speed displacement using photoelectric cells (**e**) are also examples of functional tests. Data was obtained by using the Chronojump and SmartCoach software packages

Relative to the lack of higher quality clinical trials in terms of training programs, individualized programs were mandatory when treating tendinopathies (Fig. 5). This is a well-known proposal, but it is at the same time a limitation when developing studies to be published. That is due to the effort and the need to homogenize samples. Of course, the idea of several researchers is that there is a special need to make progress in the knowledge of treating patients with tendinopathy in clinical and sports settings even when perfect homogeneity is not possible.

New data originating from basic scientific and translational investigations together with well-designed clinical trials will enhance our understanding of the origin of and identify improved treatment options for tendinopathy.

## Abbreviations

AT: Achilles tendon
CT: Computed tomography
ECRB: Extensor carpi radialis brevis
ESSKA: European Society of Sports Traumatology, Knee Surgery and Arthroscopy
HSRT: Heavy slow resistance training
MRI: Magnetic resonance image
QoL: Quality of life
RC: Rotator cuff
SSC: Stretch-shortening cycle
TERM: Tissue engineering and regenerative medicine
US: Ultra-sound
US: Ultrasound
USGET: Ultrasound-guided galvanic electrolysis technique
US-guided: Ultrasound guided
VAS: Visual analogue scale
VISA-P: Victorian Institute of Sport Assessment for Patella
